# Low Temperature Chemoresistive Oxygen Sensors Based on Titanium-Containing Ti_2_CT_x_ and Ti_3_C_2_T_x_ MXenes

**DOI:** 10.3390/ma16134506

**Published:** 2023-06-21

**Authors:** Elizaveta P. Simonenko, Ilya A. Nagornov, Artem S. Mokrushin, Sergey V. Kashevsky, Yulia M. Gorban, Tatiana L. Simonenko, Nikolay P. Simonenko, Nikolay T. Kuznetsov

**Affiliations:** 1 Kurnakov Institute of General and Inorganic Chemistry, Russian Academy of Sciences, Leninsky pr., 31, 119991 Moscow, Russia; il.nagornov.chem@gmail.com (I.A.N.); artyom.nano@gmail.com (A.S.M.); serg.kashevsky@gmail.com (S.V.K.); julia_gorban@bk.ru (Y.M.G.); egorova.offver@gmail.com (T.L.S.); n_simonenko@mail.ru (N.P.S.); ntkuz@igic.ras.ru (N.T.K.); 2 Mendeleev University of Chemical Technology of Russia, 125047 Moscow, Russia

**Keywords:** mxene, Ti_2_C, Ti_3_C_2_, chemoresistive gas sensors, 2D-nanomaterial, MOS sensor, oxygen sensor

## Abstract

The chemoresistive properties of multilayer titanium-containing Ti_2_CT_x_ and Ti_3_C_2_T_x_ MXenes, synthesized by etching the corresponding MAX phases with NaF solution in hydrochloric acid, and the composites based on them, obtained by partial oxidation directly in a sensor cell in an air flow at 150 °C, were studied. Significant differences were observed for the initial MXenes, both in microstructure and in the composition of surface functional groups, as well as in gas sensitivity. For single Ti_2_CT_x_ and Ti_3_C_2_T_x_ MXenes, significant responses to oxygen and ammonia were observed. For their partial oxidation at a moderate temperature of 150 °C, a high humidity sensitivity (T, RH = 55%) is observed for Ti_2_CT_x_ and a high and selective response to oxygen for Ti_3_C_2_T_x_ at 125 °C (RH = 0%). Overall, these titanium-containing MXenes and composites based on them are considered promising as receptor materials for low temperature oxygen sensors.

## 1. Introduction

Chemical gas sensors are increasingly in demand due to the high levels of environmental pollution caused by industry and vehicle emissions, which have a very negative impact on human health. A separate important task in this field is the express detection of oxygen, and the maintenance of its content in a sufficiently narrow range is critical for the preservation of human life, health, and performance [1]. Detection of low levels of oxygen is also required in plants that produce and use oxygen-free gas mixtures [2].

Traditionally, lambda probes have been the most commonly used oxygen sensors for determining its content in automotive emissions as an indicator of combustion efficiency. These are high-temperature electrochemical gas sensors of the YSZ-based potentiometric sensor class [3]. MOS sensors for O_2_ detection are much less widely used, although they have good prospects due to lower operating temperatures and improved sensitivity. Oxygen detection usually requires the use of relatively high temperatures (up to 400–800 °C) because the main receptor materials are wide-gap semiconductors: TiO_2_ [4,5], CeO_2_ [6,7,8], Nb_2_O_5_ [9], and mixed oxides characterized by non-stoichiometric composition and the presence of oxygen vacancies [10].

In general, reducing the detection temperature of various gaseous analytes is a clear trend in the industry, as it promotes device miniaturization (which increases device mobility and improves economic aspects) as well as sensor safety. However, there are currently no commercially available low temperature sensors on the market due to a lack of satisfactory receptor materials.

MXenes are a relatively new type of two-dimensional nanomaterials with a unique set of properties (high surface-to-volume ratio, high conductivity, high compositional diversity, possibility to vary the surface functional groups, etc.) [11,12,13,14,15]. As a result, they are widely used in many fields of science and technology: reinforcement of composite materials [16], energy generation and storage devices [17,18,19], catalysis [20,21,22], medicine [23,24,25], water purification [26,27,28], and as components of conductive coatings in optoelectronics [29,30].

The use of MXenes as receptor materials for gas sensors has been suggested previously [11,31,32,33,34]. The application of composite materials based on the combination of MXenes with semiconducting metal oxides is interesting [35], formed by partial oxidation of MXenes [36,37,38] or specially added MXenes [39,40]. For example, the high sensitivity of the Ti_3_C_2_T_x_ MXene to ammonia at room temperature has been demonstrated in studies [41,42,43] and to ethanol in studies [44,45]. The detection of acetone [46], toluene [47], and triethylamine [48] with Ti_3_C_2_T_x_ has also been established. For the compound Ti_2_CT_x_, for which information on chemosensory behavior is much more scarce, increased sensitivity has also been reported for ammonia [36,49]. The possibility of methane detection at room temperature at an irradiance of 0.85 mW·cm^−2^ has been noted [50].

While the number of publications on gas sensing with MXenes is rather high, the data on their sensitivity to oxygen are very limited. We are aware of only two papers that have reported high sensitivity in oxygen detection for Ti_2_CT_x_-based materials [49,51], which were previously carried out in our group.

The aim of the present work is to investigate the chemosensory properties of the multilayer Ti_2_CT_x_ and Ti_3_C_2_T_x_ MXenes synthesized under the NaF–HCl system and to evaluate the effect of partial oxidation at moderate temperatures on them.

## 2. Materials and Methods

### 2.1. Synthesis and Application

Reagents: powders of metallic titanium (99.9%, 0.5–100 µm, Moscow, Russia, Ruskhim), aluminum (99.2%, 30 µm, Ruskhim), graphite (99%, Technocarb, Chelyabinsk, Russia), potassium bromide KBr (99%, Moscow, Ruskhim), sodium fluoride NaF (99.9%, Moscow, Reackhim, Russia), and hydrochloric acid HCl (36%, Sigma Tech, Moscow, Russia).

The synthesis of the initial MAX phase Ti_2_AlC is described in [49,52] and the accordion-like Ti_2_CT_x_ MXene in [52]. Briefly, the initial MAX phases Ti_2_AlC and Ti_3_AlC_2_ were obtained by synthesis in protective molten salts [53,54,55], in which case KBr was used in a 1:1 mass ratio with respect to the Ti + Al + C powder mixture. To obtain Ti_2_AlC, the powders were mixed in the molar ratio *n*(Ti):*n*(Al):*n*(C) = 2:1.2:0.8. The synthesis temperature was 1000 °C and the holding time was 5 h. The synthesis of Ti_3_AlC_2_ was also carried out with an excess of aluminum and a deficiency of carbon to avoid the formation of titanium carbide impurities. The ratio of components was *n*(Ti):*n*(Al):*n*(C) = 3:1.2:1.8, the synthesis temperature was 1200 °C, and the holding time was 5 h.

The same etching system, 1.2 M sodium fluoride solution in 6 M HCl, was used to prepare the MXenes. The mass ratio of MAX phase to NaF was 1:1, the synthesis temperature was 45 ± 3 °C, the duration for Ti_2_CT_x_ was 24 h and was 48 h for Ti_3_C_2_T_x_. The obtained multilayer MXene powders were separated by centrifugation, the samples were washed with hydrochloric acid and distilled water to pH ~6, washed twice with water, and dried in a vacuum at ~80–100 °C. It should be noted that in the present work, the delamination step by tetramethylammonium or tetrabutylammonium hydroxide intercalation and the effect of intensive ultrasound were not used for the preparation of the MXene multilayer [49,51]; therefore, a characteristic accordion-like microstructure of the MXene aggregates was expected.

**Figure 1:**
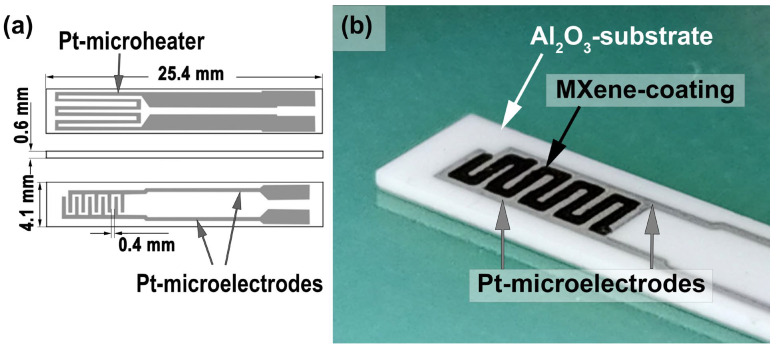
Scheme of the sensor substrate used (**a**) and photo of the applied Ti_2_CT_x_ MXene receptor layer (**b**).

MXene powders were dispersed in water (Ti_2_CT_x_) and 1-butanol (Ti_3_C_2_T_x_) in an ultrasonic bath to obtain functional inks, which were used to deposit receptor layers on specialized sensors by microplotting (Figure 1). In this case, microplotter printing was chosen due to the following advantages over other automated film-forming methods:

1. The ability to select a dispenser with the desired channel diameter, which reduces the likelihood of nozzle clogging with solid phase particles.
2. High speed and simplicity of refilling the dispenser with inks which are different in composition.
3. Variability in the film printing mechanism (discrete or continuous process), etc.

Thus, to form a Ti_2_CT_x_ film, a dispenser in the form of a glass capillary with a nozzle diameter of 150 μm was used. A dispenser was filled with the appropriate ink (about 1 μL in volume) due to capillary forces. Then, a film (a zigzag geometry that corresponds to the surface between platinum interdigital electrodes) was printed on the surface of a specialized chip. The process of film formation was carried out discretely by repeatedly touching the surface of the ink meniscus in a given area of the chip with a step of 200 μm along the X and Y axes. Thus, a Ti_2_CT_x_ film of complex geometry was obtained, consisting of sequentially overlapping pixels with a diameter of about 300 μm and a thickness of about 50 μm. The Ti_3_C_2_T_x_ film was formed on the chip surface using a similar technique. Drying was carried out in a vacuum at 100 °C and the samples were then stored in air at normal humidity.

### 2.2. Instrumentation

X-ray phase analysis of powders of the initial MAX phases, MXene, and oxide coatings was carried out using a Bruker D8 Advance X-ray diffractometer (Bruker, Billerica, MA, USA, CuK_α_ radiation, resolution 0.02° with signal accumulation in the point for 0.3 s) and DX-27mini X-ray diffractometer (Tescan s.r.o., Brno-Kohoutovice, Czech Republic, voltage–40 kV, current–12 mA). X-ray phase analysis was performed using the program MATCH! for phase identification from powder diffraction, version 3.8.0.137 (Crystal Impact, D-53227 Bonn, Germany), crystallography open database (COD). The thermal behavior of the synthesized Ti_2_CT_x_ and Ti_3_C_2_T_x_ MXene powders was studied using a combined DSC/DTA/TG analyzer SDT-Q600 (TA Instruments, 159 Lukens Dr, New Castle, DE 19720, USA) in Al crucibles in air and Ar flow (250 mL/min) with a heating rate of 10°/min and a temperature range of 25–600 °C. The following equipment was used to study the chemical composition of the surface of the powders and films and to take microphotographs of the surface of the samples: NVision 40 scanning electron microscope (Carl Zeiss, Oberkochen, Germany) and a double-beam scanning electron microscope FIB-SEM TESCAN AMBER (Tescan s.r.o., Brno-Kohoutovice, Czech Republic) using secondary electron (SE2), energy selective backscattered (EsB), in-lens detectors, and accelerating voltage 1–10 kV; transmission electron microscope (JEOL, JEM-1011, Akishima, Japan); and INCA X-MAX 80 energy dispersive X-ray (EDX) spectrometer, Oxford Instruments (Oxford, UK), with an accelerating voltage of 20 kV.

Raman spectra were recorded on an SOL Instruments Confotec NR500 Raman spectrometer (40×/0.75 objective, 532 nm laser). To avoid oxidative processes typical of MXenes when the temperature is locally raised by the laser, the power on the samples did not exceed 0.04–0.06 mW. Grating: 600. Signal accumulation time was 60 s.

**Figure 2:**
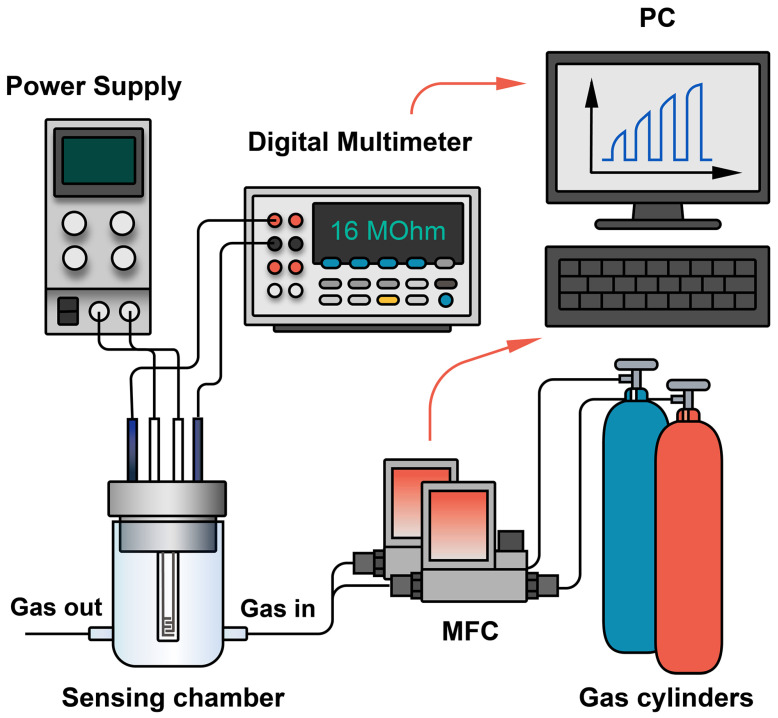
Schematic diagram of a gas detection system.

The gas-sensitive properties were measured using dedicated precision equipment [4,6]. The gas environment in the quartz cell was generated using 4 Bronkhorst gas flow controllers with maximum flow rates of 50, 100, 200, and 1000 mL/min. The temperature of the sensor cell was controlled by an integrated platinum microheater, pre-calibrated with a Testo 868 thermal imager. The resulting MXene films were tested for sensitivity to the following analytical gases: H_2_, CO, NH_3_, benzene (C_6_H_6_), acetone (C_3_H_6_O), methane (CH_4_), ethanol (C_2_H_5_OH), nitrogen dioxide (NO_2_), and oxygen (O_2_). The source of the gases analyzed was the corresponding span gas mixtures in air. Synthetic air was used as a baseline and nitrogen (99.9999%) was used for oxygen detection. The gaseous medium was created by mixing gas flows, such as baseline gas and analyte gas, in a dynamic mode. The gas flow rate was constant at 100 mL/min. The general scheme of the measuring setup is shown in Figure 2.

To measure the signal at different relative humidities (RH), we used a special device with a barbometer, and the RH of the gas mixture was controlled by a digital flow hygrometer “Excis”. The electrical resistance of the films was measured using a Fluke 8846A (6.5-digit precision multimeter) with an upper limit of 1 GΩ. Some measurements were made at room temperature (23 ± 1 °C).

The response to H_2_, CO, NH_3_, benzene (C_6_H_6_), acetone (C_3_H_6_O), methane (CH_4_), ethanol (C_2_H_5_OH), nitrogen dioxide (NO_2_), and oxygen (O_2_) was calculated by:

(1) $$S_{1}=\frac{\left|R_{BL}-R_{g}\right|}{R_{BL}}\times 100\%$$

where R_BL_—baseline resistance (nitrogen was used as the baseline for oxygen detection and synthetic air for other gases) and R_g_—resistance at a given concentration of analyte gas.

The response to humidity was calculated by:

(2) $$S_{2}=\frac{\left|R_{55}-R_{RH}\right|}{R_{55}}\times 100\%$$

where R_55_—resistance at 55% relative humidity and R_RH_—at a given relative humidity.

## 3. Results and Discussion

### 3.1. Study of the Obtained Multilayer Ti_2_CT_x_ and Ti_3_C_2_T_x_ MXene Powders

**Figure 3:**
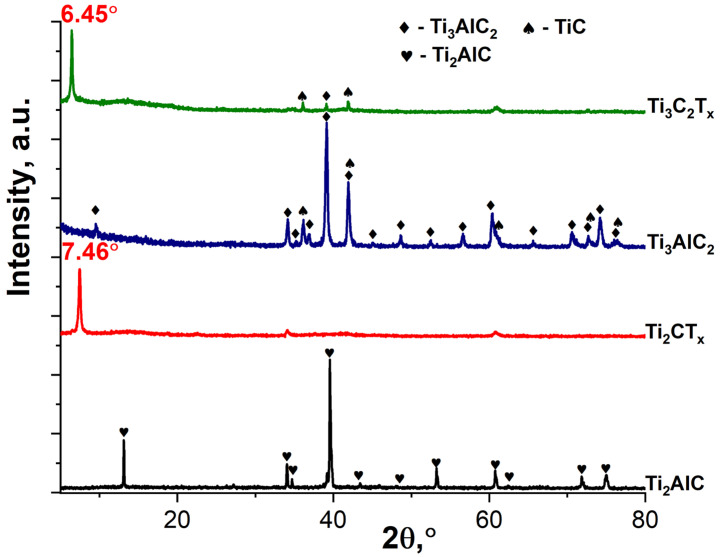
The X-ray diffraction patterns of Ti_2_AlC and Ti_3_AlC_2_ initial MAX phases and Ti_2_CT_x_ and Ti_3_C_2_T_x_ MXenes.

As can be seen from the X-ray diffraction patterns of the obtained powders (Figure 3), as a result of selective etching of the synthesized MAX phases by the NaF-HCl system, the MXene multilayers were obtained with a rather high degree of purity. For Ti_3_C_2_T_x_ powder, in contrast to Ti_2_CT_x_, the presence of small amounts of impurity TiC and residual Ti_3_AlC_2_ is noted [56]. The shift in the synthesized Ti_2_CT_x_ from the 12.88° (for Ti_2_AlC [57]) to the 7.46° position of the most intense reflex (002) for MXenes indicates an increase in the interplanar distance from 6.8 Å to 11.8 Å. For the Ti_3_C_2_T_x_ MXene, the shift in the position of this reflex from 2θ = 9.52° (for the initial Ti_3_AlC_2_) to 6.45° corresponds to a change in the interplanar distance from 9.3 to 13.7 Å.

**Figure 4:**
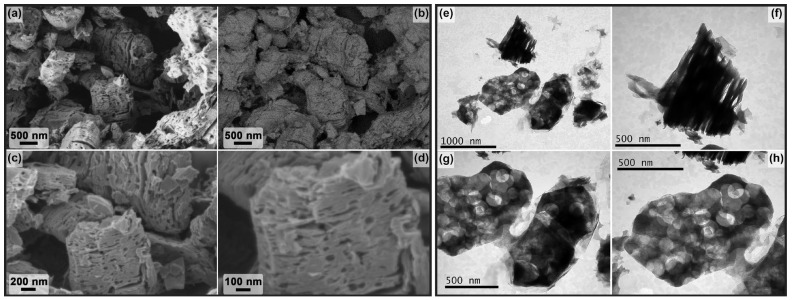
The microstructure of the obtained multilayer Ti_2_CT_x_ MXene from SEM data (**a**–**d**): in topology (**a**) and phase contrast mode (**b**) and with InLens detector (**c**,**d**), as well as from TEM data (**e**–**h**).

The microstructure and dispersion of the obtained powders of two-dimensional carbides Ti_2_CT_x_ and Ti_3_C_2_T_x_ are very different despite the same etching route and reaction parameters (composition of the etching system and temperature, only the duration was different). Thus, for the Ti_2_CT_x_ multilayer, the formation of classical accordion-like brittle clusters is observed, and their diameter and thickness vary from 500 nm to 2.5 μm. SEM micrographs (Figure 4a–d) show a high degree of defectiveness of the MXene individual layers. This is clearly seen on SEM micrographs (Figure 4e–h): they are perforated with circular holes of regular shape and diameter, predominantly 100–150 nm (rarely ~50 nm), probably inherited from the microstructure of the original MAX phase synthesized at a moderate temperature of 1000 °C. Elemental EDX analysis showed that the aluminum content is ~0.26 at.%, the *n*(F):*n*(Cl) ratio is 0.79:0.21, and the *n*(F+Cl):*n*(Ti) ratio is 1.07 (the determination of the content of surface functional groups =O and -OH, which are also present in the sample composition, is not fully accurate with this method).

**Figure 5:**
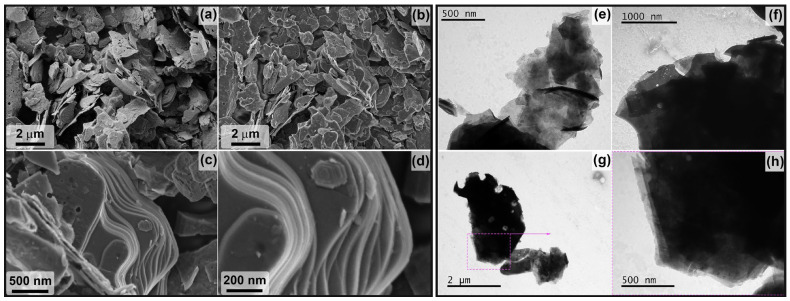
The microstructure of the obtained multilayer Ti_3_C_2_T_x_ MXene from SEM data (**a**–**d**) in topology (**a**) and phase contrast mode (**b**) and with InLens detector (**c**,**d**), as well as from TEM data (**e**–**h**).

On the other hand, the synthesized Ti_3_C_2_T_x_ MXene powder formed much denser aggregates (in agreement with the XRD data on the change in interlayer spacing) with fewer defective layers (Figure 5). The thickness of Ti_3_C_2_T_x_ MXene aggregates vary from 50 to 600 nm (which is less than this parameter for Ti_2_CT_x_) and the sheet area is much larger than that of Ti_2_CT_x_: the maximum length is mainly in the range of 2–4 μm, and less frequent are small (from 700 μm length) and large (up to 8–9 μm length) particles. Some single layer defects in the form of round holes are also observed for the Ti_3_C_2_T_x_ composition, but their number is significantly lower. The presence of TiC and Ti_3_AlC_2_ impurity particles detected by XRD, TEM, and SEM methods could not be detected, probably due to their low amount. The aluminum impurity content in the sample is 0.31 at.%, the molar ratio *n*(F):*n*(Cl) is 0.94:0.06, and the ratio *n*(F+Cl):*n*(Ti) is close to that of Ti_2_CT_x_ and is 1.03.

**Figure 6:**
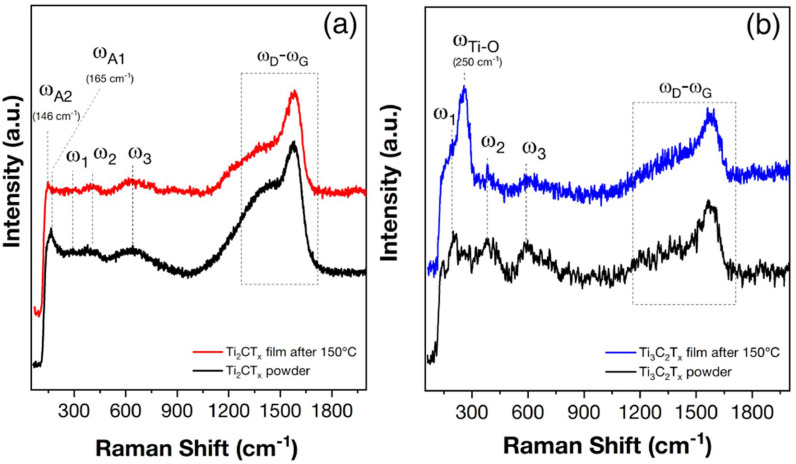
Raman spectra of the initial Ti_2_CT_x_ (**a**) and Ti_3_C_2_T_x_ (**b**) powders as well as their films after heating in the sensor cell to 150 °C.

Figure 6 shows the Raman spectra of the original Ti_2_CT_x_ and Ti_3_C_2_T_x_ MXene powders. As can be seen for the Ti_2_CT_x_ MXene powder, three Ti_2_CT_x_ MXene eigenmodes are observed in the Raman spectrum: ω_1_–ω_3_ at 290, 402, and 635 cm^−1^, which correlate well with the literature data [58]. In addition, two broadened intense peaks, ω_D_ and ω_G_, are present in the 1200–1600 cm^−1^ region, related to the D- and G-bands of carbon in the MXenes [59]. The medium intensity mode ω_A1_ at 165 cm^−1^ probably refers to an oxygen non-stoichiometric Ti-O bond [60] (possible product of minor oxidation in aqueous medium during extraction and purification of MXenes). For the obtained Ti_3_C_2_T_x_ powder, the three intrinsic modes of this substance appeared, ω_1_–ω_3_ at 200, 380, and 590 cm^−1^, whose position and shape correlate well with the literature data [61]. As for Ti_2_CT_x_, two broadened intense peaks, ω_D_ and ω_G_, are present in the 1200–1600 cm^−1^ region for Ti_3_C_2_T_x_ [59].

**Figure 7:**
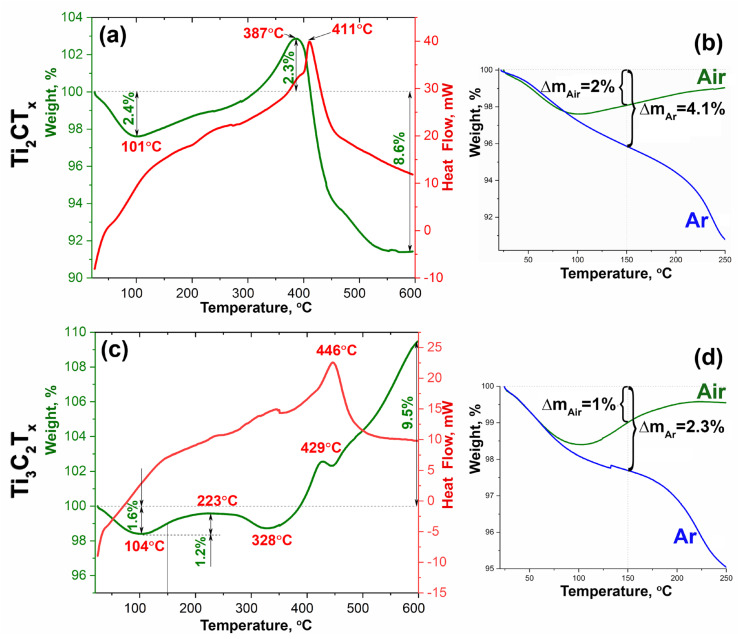
The DSC (red) and TGA (green) curves obtained by heating Ti_2_CT_x_ (**a**,**b**) and Ti_3_C_2_T_x_ (**c**,**d**) powders in air, as well as the corresponding superposition of the TGA curves in air (green) and argon (blue) in the temperature range 20–250 °C (**b**,**d**).

The thermal behavior of MXene powders in airflow and argon in the temperature range 20–600 °C was studied (Figure 7). As can be seen, the consequence of the differences in the microstructure of Ti_2_CT_x_ and Ti_3_C_2_T_x_ is a significant difference in the sorption properties of these powders: the mass loss in an argon atmosphere at 250 °C was 9.2% for the first sample and 5.0% for the second. However, both samples are highly reactive in the oxidation process—a corresponding increase in mass is already observed at temperatures above 100 °C.

In particular, for Ti_2_CT_x_ in an air flow at a temperature below 101 °C, there is a 2.4% mass loss caused by the desorption of water molecules from the surface and from the interlayer space of the MXene accordion-like particles. The subsequent increase in mass may be due to the onset of the oxidation process, which proceeds simultaneously with the ongoing desorption and detachment of surface functional groups. At temperatures >387 °C a loss of mass is again observed for Ti_2_CT_x_, which may indicate the prevalence of MXene destruction processes under these conditions; a sufficiently large air flow (250 mL/min) may also carry away two-dimensional nanoparticles of decomposition products detached by heating the sample, accompanied by intense gas emission.

For the Ti_3_C_2_T_x_ sample, due to the less gas-permeable structure of the layer stacks, against the background of manifest oxidation (expressed as an increase in mass at temperature >104 °C), the degradation processes are more visible: at low temperature there is a desorption of water molecules, which is superimposed at higher temperatures (probably in the range 150–350 °C) by a process of detachment of surface functional groups -OH, -F, and -Cl.

In general, it can be observed that for both MXene titanium powders obtained, the oxidation, as determined by DSC/TA data, starts at extremely low temperatures (around 70–100 °C) when heated in an air flow. This is particularly evident when the loss of mass curves for heating in air and argon atmospheres are superimposed (Figure 7b,d). Due to the established fact in experiments to study the influence of partial oxidation of Ti_2_CT_x_ and Ti_3_C_2_T_x_ on their chemosensory properties, the temperature of heating in air flow did not exceed 150 °C in order to avoid complete oxidation of the samples.

### 3.2. Study of Multilayer Ti_2_CT_x_ and Ti_3_C_2_T_x_ MXene Coatings after Partial Oxidation in a Sensor Cell

In order to improve the chemosensory properties of Ti_2_CT_x_ and Ti_3_C_2_T_x_ MXenes by forming nanocomposites with nanoparticles of semiconducting titanium oxide (which is mentioned in most literature sources [11,51,62,63,64]), the process of their partial oxidation at temperature 150 °C was carried out directly in the measuring cell (to control the change of the coating resistance). The temperature was increased in steps (50, 75, 100, 125, and 150 °C) with an exposure time of 10 min at each intermediate step. It was found that the resistance of the MXene coatings decreased on heating, indicating the semiconducting nature of the conductivity, which is rare for MXene. During baking, a slow increase in resistance is observed at certain temperature stages, which is related to its oxidation and the formation of a Schottky barrier at the interface.

**Figure 8:**
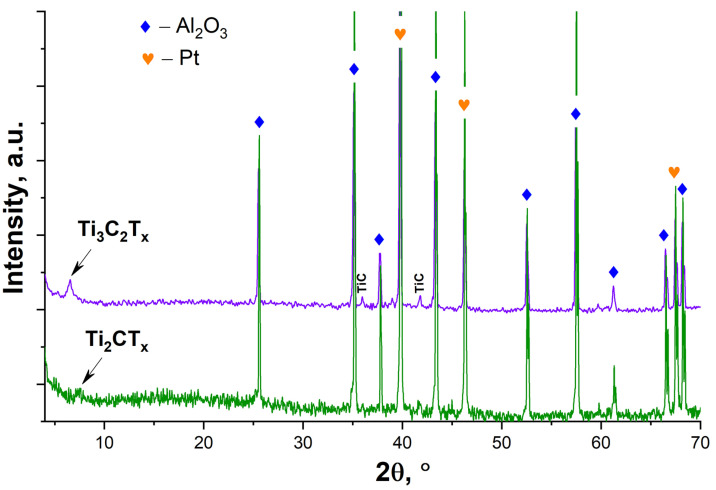
The X-ray diffraction patterns of Ti_2_CT_x_ and T_3_C_2_T_x_ coatings on the sensor surface after partial oxidation at a temperature of 150 °C.

Analysis of the X-ray diffraction patterns of the Ti_2_CT_x_ and Ti_3_C_2_T_x_ coatings on the sensing element has shown (Figure 8) that the reflections (002) characteristic of MXenes were preserved against the background of intense Al_2_O_3_ (substrate) [65] and platinum (electrodes) [66] reflections due to heating in air. It is difficult to detect a significant shift in the position of this reflection due to its low intensity. No reflections of the oxidation products (titanium oxides) were found either, which may be due to their small amount and also due to the fact that their position overlaps with the intense reflections of the substrate.

According to Raman spectroscopy (Figure 6a), after in situ heating of the Ti_2_CT_x_ MXene film to 150 °C, the characteristic initial MXene band set (ω_1_–ω_3_ and ω_D_–ω_G_) is preserved, but there is a shift in the ω_A1_ peak from 165 to 145 cm^−1^ (ω_A2_), i.e., it approaches the ω_A2_ mode position of TiO_2_ with anatase structure (145 cm^−1^) [60]. For the Ti_3_C_2_T_x_ MXene, the characteristic initial MXene band set (ω_1_–ω_3_ and ω_D_–ω_G_) also persists and an intense ω_Ti-O_ peak appears at 250 cm^−1^, which refers to the MXene oxidation products, as seen in Figure 6b. The appearance of the ω_Ti-O_ band could refer to the multiphoton signal of the rutile TiO_2_ phase [60,67]. This variant is unlikely as these processes appear as low intensity bands in the Raman spectra of rutile. The most intense modes of rutile are E_g_ and A_1g_ at ~445 and 610 cm^−1^, which are not observed in the spectra of partially oxidized Ti_3_C_2_T_x_. The second assignment is to the Ti_2_O_3_ phase, characterized by the most intense signal at 247 cm^−1^ [68], which agrees well with the data in Figure 7b. Thus, it is shown that heating to 150 °C in an air flow preserves the MXene structure for both Ti_3_C_2_T_x_ and Ti_2_CT_x_ but the characteristic modes of the oxidation products appear, in particular TiO_2_ with anatase structure for Ti_2_CT_x_ and Ti_2_O_3_ for Ti_3_C_2_T_x_.

**Figure 9:**
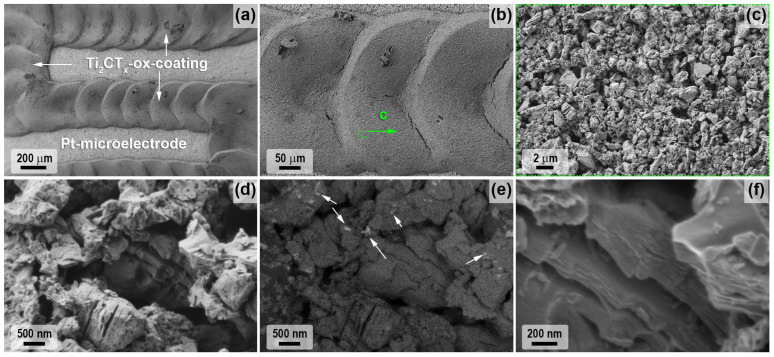
The microstructure of the multilayer Ti_2_CT_x_ MXene coating after partial oxidation at 150 °C according to SEM data: topology study mode (**a**–**d**) and phase contrast (**e**). Arrows show examples of oxidized areas) and with InLens detector (**f**).

SEM data analyzing microregions of the partially oxidized Ti_2_CT_x_ MXene coating indicate that no fundamental changes in the microstructure of the accordion-like aggregates occur under these conditions (at a relatively low temperature of 150 °C and holding time) (Figure 9). However, the phase contrast image (Figure 9c) shows that nanosized phenocrysts, mostly 30 to 200 nm in size, of oxidized phase, presumably anatase, are present on the lateral surfaces of the aggregates. The ratio *n*(F+Cl):*n*(Ti) halved from 1.07 to 0.53. This may be due to the detachment of these surface functional groups during heating, which were replaced by =O substituents, which may partially transform into hydroxo groups after storage in a humid environment.

**Figure 10:**
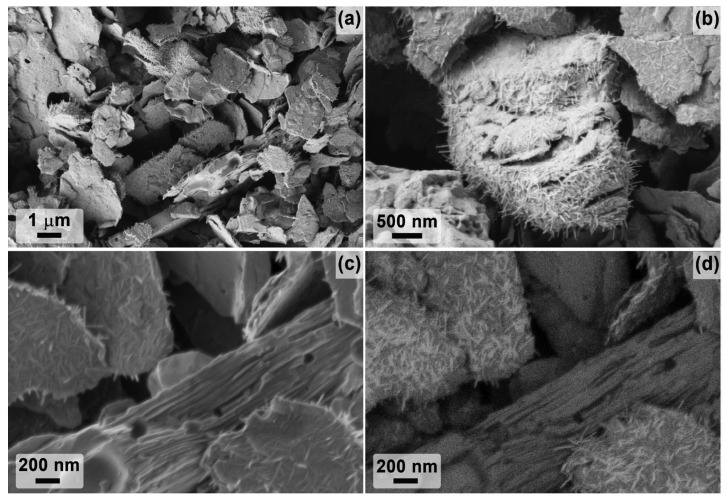
The microstructure of the multilayer Ti_3_C_2_T_x_ MXene coating after partial oxidation at 150 °C according to SEM data: topology study mode (**a**) with InLens detector (**b**–**d**).

The change in the Ti_3_C_2_T_x_-coating microstructure after its low-temperature partial oxidation was also studied by SEM (Figure 10). It was found that in this case there were no significant changes in the size and shape of MXene aggregates, only that needle formations of less conductive phases (apparently titanium oxide) with diameters of 10–30 nm and lengths 100–200 nm appeared on the intergrowth surface. Thus, titanium oxide needles were mainly formed on the MXene plane, and they are practically not found on the edges of the stacks. The EDX analysis data show that the molar ratio *n*(F):*n*(Cl) remained unchanged after oxidation 0.94:0.06 (in contrast to the sample Ti_2_CT_x_-ox), and the ratio *n*(F+Cl):*n*(Ti) also decreased by half to 0.56.

### 3.3. Chemoresistive Properties of Ti_2_CT_x_ and Ti_3_C_2_T_x_ Coatings Deposited by Microplotting

In the first step of the chemoresistive measurements, the sensitivity to a wide group of analyte gases (100 ppm C_3_H_6_O, C_2_H_5_OH, CO, NH_3_, C_6_H_6_, NO_2_, 1000 ppm H_2_, CH_4_, and 10% O_2_) at room temperature (RT) was comprehensively investigated for the obtained multilayer Ti_2_CT_x_ and Ti_3_C_2_T_x_ MXenes. Due to the different sensitivity to humidity, measurements for the Ti_3_C_2_T_x_ MXene were carried out at 0% RH and for the Ti_2_CT_x_ MXene at RH = 55%.

Thus, for the obtained Ti_2_CT_x_ MXene, when initially measured at RT and RH = 0%, the response to various gases was found to be markedly sensitive to 10% O_2_ (S = 10%) and 100 ppm ammonia (S = 2.5%). The response to 100 ppm C_3_H_6_O, C_2_H_5_OH, CO, C_6_H_6_, 1000 ppm H_2_, and CH_4_ did not exceed 1.3%. The measurement of the response to 100 ppm NO_2_ resulted in a very high and irreversible response (>70%), which led to a measured resistance increase of more than 1 GΩ; therefore, no further measurements were possible at RT and RH = 0%. This situation may be due to irreversible chemical reactions between NO_2_ and MXene (including oxidation of the latter).

**Figure 11:**
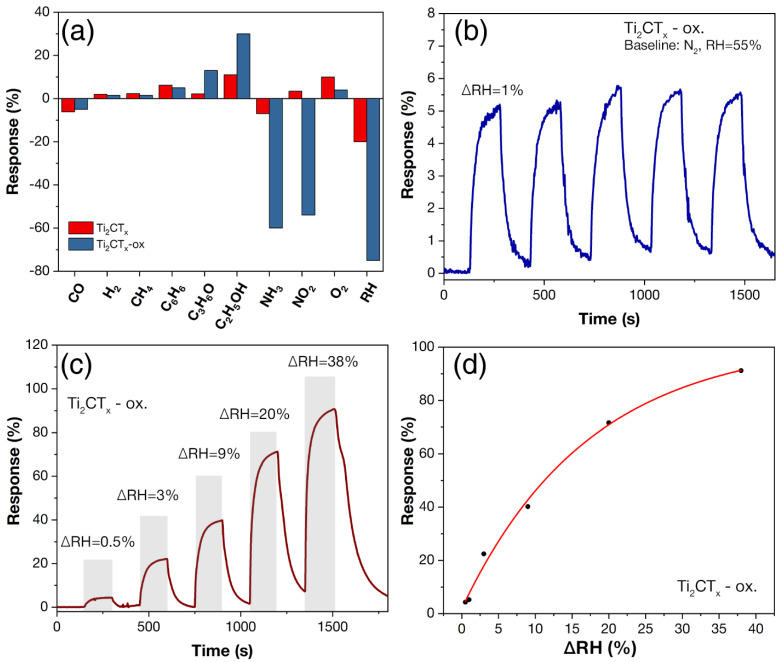
The gas-sensing properties of the Ti_2_CT_x_ MXene before and after heating to 150 °C (Ti_2_CT_x_-ox). The selectivity histogram with responses to different gas concentrations (100 ppm C_3_H_6_O, C_2_H_5_OH, CO, NH_3_, C_6_H_6_, NO_2_, 1000 ppm H_2_, CH_4_, 10% O_2,_ and humidity change ΔRH by 20%, RT and RH = 55% conditions) (**a**); repeatability of response to RH change by 1% (ΔRH) (**b**); responses to RH change by 0.5–38% (**c**); and their dependence on ΔRH (**d**). (**b**–**d**) shows the data for the Ti_2_CT_x_-ox sample, where nitrogen at RH = 55% was used as the baseline. The “–” sign in the diagram (**a**) indicates a decrease in electrical resistance, and the “+” sign indicates an increase during gas admission.

The Ti_2_CT_x_ MXene was also found to be highly sensitive to humidity. With increasing RH, a significant decrease in electrical resistance was observed, down to a value below the measurement limit. Therefore, the gas sensitivity of this compound was further investigated at RH = 55%, as under these conditions it was possible to significantly reduce the base resistance to the measured values. As can be seen from Figure 11a, the highest response was found to CO, C_6_H_6_, C_2_H_5_OH, NH_3_, and O_2_ with 6, 6, 11, 7, and 10%, respectively. The response to other gases did not exceed 3.4%. The high response (20%) to a 20% change in relative humidity is also noteworthy (ΔRH).

**Figure 12:**
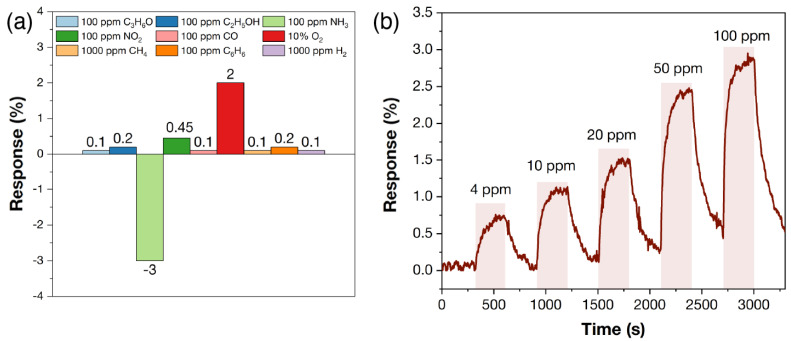
The gas-sensing properties of Ti_3_C_2_T_x_ MXene at RT and RH = 0%. The histogram of selectivity with responses to different gas concentrations of 100 ppm C_3_H_6_O, C_2_H_5_OH, CO, NH_3_, C_6_H_6_, NO_2_, 1000 ppm H_2_, CH_4_, and 10% O_2_ (**a**) and responses to 4–100 ppm NH_3_ (**b**). The “–” sign in the diagram (**a**) indicates a decrease in electrical resistance, and the “+” sign indicates an increase during gas admission.

The histogram of selectivity consisting of responses to 100 ppm C_3_H_6_O, C_2_H_5_OH, CO, NH_3_, C_6_H_6_, NO_2_, 1000 ppm H_2_, CH_4_, and 10% O_2_ at RT is shown in Figure 12a for the Ti_3_C_2_T_x_ MXene coating. The Ti_3_C_2_T_x_ MXene shows an increased sensitivity to ammonia and oxygen with responses of 3 and 2%, respectively. The responses to other gases did not exceed 0.45% (for NO_2_). High sensitivity to ammonia is typical of both Ti_2_CT_x_ [36,69] and Ti_3_C_2_T_x_ [41,43,70]. It is worth noting that a relatively high sensitivity to oxygen was observed: at 10%, O_2_ the response was ~2%. Figure 12b shows the response to 4–100 ppm NH_3_: as the ammonia concentration increases from 4 ppm to 100 ppm, and an increase in response from 0.7 to 3% is observed. The minimum detection limit is low at 4 ppm, which is in the MAC range [71]. The response time and signal recovery time (t_90_) were 177–174 and 231–236 s, respectively, at increases of 4 ppm to 100 ppm NH_3_.

### 3.4. Chemoresistive Properties of Ti_2_CT_x_ and Ti_3_C_2_T_x_ Coatings after Partial Oxidation in the Sensor Cell

The sensory properties of the Ti_2_CT_x_ partially oxidized sample (sample Ti_2_CT_x_-ox) were also investigated. Figure 11 shows a selectivity diagram with responses to different gases as well as responses to changes in relative humidity (ΔRH) compared to the initial MXene (RT, RH = 55%). It is shown that after partial oxidation, a significant change in the sensitivity of Ti_2_CT_x_ to some analyte gases was observed: the response to 100 ppm acetone, ethanol, ammonia, and NO_2_ increased from 2.2 to 13%, from 11 to 30%, from 7 to 60%, and from 3.4 to 54%, respectively. There was also a significant increase in sensitivity to changes in humidity: for ΔRH = 20% the response increased from 20 to 75%.

Figure 11b shows the reproducibility of the signal at ΔRH = 1%: a high and reproducible response is observed when several cycles of changing the gas environment are performed. When ΔRH is changed from 0.5 to 38%, the response increases significantly from 4.3 to 91.2% (Figure 11c). The dependence of the response on ΔRH is well described (R^2^ = 99.5%) by the power function equation (Figure 11d). For the minimum detection limit (ΔRH = 0.5%) obtained in the present work, the response is 4.3%, which is quite high for room temperature sensors. The reason for the increased sensitivity to moisture and MXene typical analytes could be an increase in the adsorption area when the accordion-like Ti_2_CT_x_ aggregates are loosened by surface oxidation, as well as a change in the composition of their surface functional groups. The response time and signal recovery time (t_90_) were 62–50 and 24–190 s, respectively, with ΔRH being changed from 0.5 to 38%.

For the second Ti_3_C_2_T_x_ partially oxidized at 150 °C (sample Ti_3_C_2_T_x_-ox), no significant improvement in the chemosensory properties at RT was found; therefore, the chemosensory properties (including oxygen detection) at higher temperatures in the range 25–125 °C were investigated.

For example, the response to 10% O_2_ was determined as a function of the detection operating temperature in order to determine the optimum detection temperature. As the detection temperature is increased, a significant increase in the oxygen response is observed, with a maximum at 125 °C. Experiments at higher detection temperatures were not carried out to avoid further oxidation of the Ti_3_C_2_T_x_ MXene. Therefore, the chemoresistive properties of the Ti_3_C_2_T_x_-ox sample were investigated in more detail at 125 °C.

**Figure 13:**
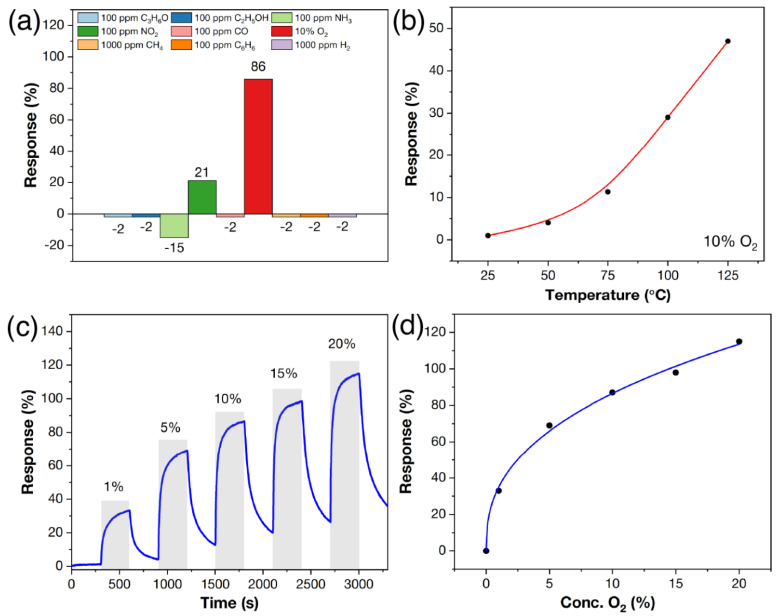
The gas-sensing properties of the Ti_3_C_2_T_x_-ox sample. Selectivity histogram with responses to different gas concentrations of 100 ppm C_3_H_6_O, C_2_H_5_OH, CO, NH_3_, C_6_H_6_, NO_2_, 1000 ppm H_2_, CH_4_, and 10% O_2_ at 125 °C operating temperature (**a**); dependence of response to 10% O_2_ on detection temperature (**b**); responses to 1–20% O_2_ (**c**); and dependence of response to O_2_ concentration at 125 °C detection temperature (**d**). In all cases, measurements were carried out at RH = 0%. The “–” sign in diagram (**a**) indicates a decrease in electrical resistance, and the “+” sign indicates an increase during gas admission.

Figure 13a shows the histogram of selectivity compiled from the responses to different gas concentrations at 125 °C. It can be seen that for the Ti_3_C_2_T_x_-ox sample, the response to most gases is significantly higher than that of the initial non-oxidized Ti_3_C_2_T_x_ MXene sample measured at room temperature. In this case, the response to oxygen is significantly higher than that to other gases and is 86% for 10% O_2_. A remarkable sensitivity is also observed to 100 ppm NO_2_ (21%) and NH_3_ (15%), with the response for other gases not exceeding 2%. A study of the response to 1–20% O_2_ at 125 °C (Figure 13c) shows that the responses are bell-shaped, and as the O_2_ concentration increases from 1 to 20%, the responses increase from 33% to 115%. The dependence of the response on oxygen concentration is well described (R^2^ = 99.2%) by a power function of the Freundlich isotherm equation (Figure 13d), which is typical of medium- and low-temperature chemoresistive gas MOS sensors to oxygen [9]. The response time and signal recovery time (t_90_) were 158–171 and 151–203 s, respectively, at increases of 1–20% O_2_.

A possible reason for the high sensitivity to oxygen of the Ti_3_C_2_T_x_-ox sample is the formation of a non-stoichiometric oxygen Ti_2_O_3_ phase (Figure 10) on the surface of the MXene aggregates, identified by Raman spectroscopy (Figure 6). This means that the emergence of an enhanced response at relatively low temperatures for oxygen gas sensors 125 °C can be contributed by the formation, as a result of partial oxidation, of Ti_3_C_2_T_x_ nanodispersed and non-aggregated needle-shaped nanoparticles of semiconductor titanium oxide phase, as well as the corresponding growth of the surface available for gas adsorption.

Classical views of the oxygen-sensing mechanism of MOS gas sensors, such as TiO_2_, CeO_2_, Nb_2_O_5_, etc., use a model describing surface reactions between oxygen and various defects caused by non-stoichiometric composition, mainly oxygen vacancies ($V_{o}^{••}$) [3]. It is likely that in the present work, the contribution to the enhanced oxygen response in terms of the interaction of TiO_x_ particles on the MXene surface aggregates also plays a significant role. This is further evidenced by the change (growth) in the response value with increasing detection temperature, which is typical for MOS sensors but not for MXenes. It is known that titanium oxides, especially in the nanodispersed state, can have strong oxygen non-stoichiometry, and titanium cations can be both in the lattice nodes in different oxidation states (from +II to +IV) and in the inter-nodal position, forming titanium intercalations [72]. As a consequence, various defects such as oxygen vacancies ($V_{o}^{••}$) can form. The following equilibrium reaction occurs when the oxygen concentration is introduced or increased [4]:

(3) $$V_{o}^{••}+\frac{1}{2}O_{2}+2e^{–}↔O_{o}^{x}$$

This causes oxygen from the gas phase to be incorporated into the oxygen vacancy, which takes electrons from the conduction band of the material and leads to an increase in resistance, i.e., a chemoresistive response.

The formation of a Schottky barrier at the interface and the formation of titanium defects as a result of oxidation of the MXenes in their composition may also be the cause of the additional increase in response, as reported in the literature [38,62,63].

## 4. Conclusions

A study of the gas sensitivity of the Ti_2_CT_x_ and Ti_3_C_2_T_x_ multilayer MXene coatings obtained by microplotting showed significant differences in their sensing characteristics. Thus, for Ti_2_CT_x_ at room temperature and RH = 0%, sensitivity to oxygen and ammonia was observed, but an irreversible interaction with NO_2_ (100 ppm) resulted in a sharp increase in resistance, preventing further experiments in dry gases. A study carried out at RH = 55% showed a high sensitivity to humidity, ethanol, oxygen, ammonia, and CO. For the Ti_3_C_2_T_x_ MXene, increased sensitivity to ammonia and oxygen was noted when detected at RH = 0% and room temperature, but the responses were low, not exceeding 3%.

In order to improve MXene chemosensory properties, their partial oxidation was carried out directly in the measuring cell at a temperature of 150 °C. The complexity of physicochemical analysis methods shows that under these conditions, the MXene phase is preserved and a small amount of anatase phase appears for Ti_2_CT_x_ and Ti_2_O_3_ for Ti_3_C_2_T_x_. Changes in the composition and microstructure of the receptor materials caused a significant change in their gas sensitivity:

1. For the partially oxidized accordion-like Ti_2_CT_x_ Mxene, there is a sharp increase in sensitivity to humidity and a decrease in the existing sensitivity to oxygen (measurements performed at RT and RH = 55%). The selectivity towards other analytes also changes: the highest sensitivity of the initial Ti_2_CT_x_ to 100 ppm ethanol (11% response) for the Ti_2_CT_x_-ox sample is replaced by high responses to 100 ppm NH_3_ (S = 60%) and NO_2_ (S = 54%).
2. The Ti_3_C_2_T_x_ multilayer modified with needle-shaped Ti_2_O_3_ particles retains high sensitivity to oxygen in dry air, but this requires a slightly higher detection temperature; the optimum operating temperature was found to be 125 °C, at which the response to 10% O_2_ is 86%. The response to 1% oxygen under these conditions is 33%. Significant sensitivity was also observed to 100 ppm NO_2_ (21%) and NH_3_ (15%), with responses to other gases not exceeding 2%.

In conclusion, titanium-containing MXenes and composites based on them are promising receptor materials for low temperature oxygen sensors. Partial oxidation of these MXenes leads to a decrease in sensitivity to O_2_ (at 55% RH) for Ti_2_CT_x_. Partial oxidation of Ti_3_C_2_T_x_, on the other hand, requires an increase in operating temperature to 75–125 °C to obtain high oxygen responses in a dry gas environment.

## Data Availability

Not applicable.
